# Supplementary material for: Adjuvant Chemotherapy in Older Patients with Gastric Cancer: A Population-Based Cohort Study
Source: Cancers (Basel). 2023 Jul 25;15(15):3768. doi: 10.3390/cancers15153768 (PMC10417693; doi:10.3390/cancers15153768)
Supplement: Supplementary file 1 [file cancers-15-03768-s001.zip › cancers-2423017-supplementary.pdf]

Supplementary Table S1. Sensitivity analysis of survival outcomes

Outcomes	HR	95%CI	*P*-value
Overall survival	0.75	(0.71, 0.79)	<0.001 *
Cancer-specific survival	0.83	(0.76, 0.90)	<0.001 *

Abbreviation: HR = hazard ratio; CI = confidence interval. * Significant at 0.05 level by Cox proportional hazard regression.

Supplementary Table S2. Multivariable logistic regression of overall survival

Variables	Overall survival		
	OR	95%CI	*P*-value
Chemotherapy	0.52	(0.41,0.66)	<0.001*
Age	1.06	(1.04,1.09)	<0.001*
Male (ref: female)	1.35	(1.04,1.75)	0.022*
WBC	1.03	(0.95,1.11)	0.448
Hb	0.73	(0.61,1.08)	0.087
Platelet	1.00	(0.99,1.00)	0.107
Neutrophil	1.01	(0.95,1.08)	0.725
Lymphocyte	0.82	(0.66,1.02)	0.069
ALP	1.00	(0.99,1.00)	0.214
eGFR	1.00	(0.99,1.00)	0.914
Total bilirubin	1.00	(0.98,1.01)	0.523
Albumin (ref: 30-50 g/l)	1.47	(1.08,2.04)	0.017*
eGFR (ref: ≤60 $ml/min/1.73m^2$)	0.84	(0.24,2.92)	0.782
CCI (ref: <8)	0.90	(0.54,1.50)	0.676
Coronary heart disease	0.80	(0.41,1.55)	0.502
Heart failure	2.01	(0.57,7.01)	0.275
Stroke	1.99	(1.01,3.93)	0.048*
Atrial fibrillation	0.49	(0.18,1.37)	0.174
Diabetes	1.23	(0.82,1.83)	0.323
Liver disease	0.74	(0.31,1.77)	0.500
Radiotherapy	0.85	(0.14,1.46)	0.655

Abbreviation: OR = odds ratio; CI = confidence interval; CCI= Charlson Comorbidity Index; WBC= white blood cell; Hb = Hemoglobulin; ALP= Alkaline Phosphatase; eGFR = estimated glomerular filtration rate; CrCl =Creatinine Clearance.
* Significant at 0.05 level by multivariable logistic regression.
